# A Modular Mind? A Test Using Individual Data from Seven Primate Species

**DOI:** 10.1371/journal.pone.0051918

**Published:** 2012-12-19

**Authors:** Federica Amici, Bradley Barney, Valen E. Johnson, Josep Call, Filippo Aureli

**Affiliations:** 1 Department of Comparative and Developmental Psychology, Max Planck Institute for Evolutionary Anthropology, Leipzig, Germany; 2 Department of Mathematics and Statistics, Kennesaw State University, Kennesaw, Georgia, United States of America; 3 Department of Biostatistics, M.D. Anderson Cancer Center, Houston, Texas, United States of America; 4 Instituto de Neuroetologia, Universidad Veracruzana, Xalapa, Veracruz, Mexico; 5 Research Centre in Evolutionary Anthropology and Palaeoecology, Liverpool John Moores University, Liverpool, United Kingdom; German Primate Centre, Germany

## Abstract

It has long been debated whether the mind consists of specialized and independently evolving modules, or whether and to what extent a general factor accounts for the variance in performance across different cognitive domains. In this study, we used a hierarchical Bayesian model to re-analyse individual level data collected on seven primate species (chimpanzees, bonobos, orangutans, gorillas, spider monkeys, brown capuchin monkeys and long-tailed macaques) across 17 tasks within four domains (inhibition, memory, transposition and support). Our modelling approach evidenced the existence of both a domain-specific factor and a species factor, each accounting for the same amount (17%) of the observed variance. In contrast, inter-individual differences played a minimal role. These results support the hypothesis that the mind of primates is (at least partially) modular, with domain-specific cognitive skills undergoing different evolutionary pressures in different species in response to specific ecological and social demands.

## Introduction

One of the most consistent findings from individual-variability research focusing on human cognitive abilities and disabilities is that diverse cognitive processes interrelate even when they have little in common (see [1] for a review). As first evidenced by Spearman [2], individual performance in humans positively correlates across different cognitive domains, with substantial variation accounted for by a single factor. This single factor accounting for most of the variance across domains has been labelled G, or general factor of intelligence, and it appears to be related to a variety of psychological, social, biological and genetic factors, including brain volume and amounts of grey and white matter [3], [4]. More recently, the existence of a general factor qualitatively and quantitatively analogous to G has also been suggested in mice [5]–[12] (but see [13]). In these studies, a general factor accounted for 23–44% of the variance in performance across different cognitive domains, similarly to what was reported for humans with G accounting for about 40% of the total variance [1].

Some scientists have challenged the existence of G by claiming that the mind consists either entirely or largely of specialized, independently evolving modules [14]–[18]. Experimental evidence in humans supporting this view ranges from precocious development in some specific domains, to dissociable damage to many (but not all) individual systems (reviewed in [14]). In mammals, evidence of mosaic evolution in brain organization (i.e. different cognitive skills having evolved independently in different lineages) provides indirect support to this view [19], [20]. From a theoretical point of view, the modularity of mind can be explained by domain-specific cognitive skills reflecting adaptations to specific socio-ecological problems aimed to increase fitness in taxon-typical environment [21]–[23]. In this respect, the mind would consist of several specialized modules, each independently evolved to solve specific problems [15], [23]–[25].

An intermediate view considers the existence of G being compatible with the existence of independent domain-specific cognitive skills [1], [26]. In humans, some properties of the brain, such as the amount of grey matter and the neuronal speed of transmission, have a general effect on different brain regions, leading to correlations across performance in different domains even if cognitive processes are localized in discrete regions [27]–[31]. Because the link between brain measures and cognitive skills is controversial (e.g. [32], [33]), experimental support of this view is still needed. Indeed, as soon as a reversal of performance in two different domains between two species is found (e.g. species 1 does better on task A than species 2, but species 2 does better on task B than species 1), the existence of domain-specific cognitive skills can be claimed. Since such events are rather common (e.g. [34]), it seems that domain-specific abilities must logically exist. Then, the real issue is how to best capture correlations across tasks and estimate *how much* of the variance a common factor (G) can explain.

The question about whether and to what extent minds are modular has been recently addressed in non-human primates. These studies mainly investigated whether some individuals or taxa consistently perform better than others across different cognitive domains. For example, Deaner and colleagues [26] used a hierarchical Bayesian model to compare the performance of 24 genera in several cognitive domains ranging from tool use to memory and inhibition. In their study, genera performing better in one domain performed better in many other domains, with a single general factor accounting for approximately 85% of the variance. Consequently, Deaner and colleagues [26] concluded that primate taxa may differ in a general factor analogous to the human G.

Similarly, Reader and colleagues [35] compared 62 primate species across a variety of domains including social learning, tactical deception, tool use, innovation and extractive foraging. Their results evidenced a single factor explaining over 65% of the variance across domains and covarying with the general factor from Deaner and colleagues [26]. The strong correlation between distinct measures of primate cognitive performance led the authors to conclude that cognitive skills in primates are not independent. However, their study could not rule out that the primate mind consists of distinct modules which have partially coevolved, nor that modularity exist outside of the tested domains [35], [36]. In particular, independent cognitive skills might have undergone correlated evolution by being subject to the same selective pressures [26], [37]. For example, the cognitive requirements of group living might be linked to both enhanced social coordination and analogical reasoning (e.g. [38], [39]). Consequently, some taxa might perform better across different domains simply because they possess most of the few distinct but co-evolved skills, each of which is required for such domains [26], [35].

In a recent study, Schmitt and colleagues [40] experimentally tested long-tailed macaques (*Macaca fascicularis*) and olive baboons (*Papio anubis*) in a series of tasks on physical and social cognition ranging from the understanding of spatial and causal relations to the understanding of others’ intentions. They found that interspecific differences could not be explained by a domain-general factor, but rather at the domain-specific level. Schmitt and colleagues [40] explained the differences with previous studies in terms of the wider number of tasks and the same methods used for all subjects. This last aspect is especially important, as *direct* comparison of different species is fundamental to better understand how cognitive skills are distributed across species (e.g. [41]). Other studies have addressed the topic of mind modularity by focusing on inter-individual variability within a species. If some individuals perform better in some domains, whereas other individuals do so in other domains, the notion of modular minds would be supported; on the contrary, if the same individuals perform consistently better across all domains, a general factor would be supported [26], [42], [43]. Herrmann and colleagues [44], for example, used 15 physical and social domains to investigate individual differences in the cognitive skills of human children and chimpanzees (*Pan troglodytes*). In chimpanzees, one single factor was not sufficient to account for the variance in performance across the different domains, with performance being best explained by one factor accounting for performance on spatial domains, another factor accounting for performance on two physical and two social domains, and no other factor accounting for performance in the remaining domains [44]. In contrast, Banerjee and colleagues [45] found evidence for a general factor when testing 22 cotton-top tamarins (*Saguinus oedipus*) on different cognitive skills, including inhibition and memory domains. Contrasting results have also been reported for species other than primates, with a positive association across individual performance in different cognitive domains being shown in honey bees (*Apis mellifera*: [46]), but not in song sparrows (*Melospiza melodia*: [47]), and only partially in satin bowerbirds and mice (*Ptilonorhynchus violaceus*: [48]; mice: [8]).

The relative importance of G might be inflated by researchers choosing experimental tasks which seem to require cognitive skills from different domains, but are not sufficiently different from one another. If tasks are even slightly similar, it is not surprising that the performance across them is correlated. For example, although mice are usually reported to have been tested on a battery of tasks “tapping diverse cognitive demands”, the administered tasks are basically all spatial (e.g. [7], p. 88). Another problem with studies investigating domain-specific and general factors across species is that data used in the analyses are often not at the individual level. Analyses using data published in various articles, for example, can include a large variety of tasks from different domains, but they lose much information by using rank data at the genus or species level, instead of data at the individual or trial level (e.g. [26]). Consequently, the existence of individual-level effects cannot be determined, the importance of G might be inflated, and the existence of domain-specific effects might be harder to detect. Moreover, these analyses rely on different studies which probably have not used the same testing procedures for every species, so that these analyses might detect inter-specific differences simply caused by methodological differences.

In this study, we aimed to explore the existence of domain-specific versus general factors accounting for the variance in performance across different cognitive domains in seven primate species. In particular, we investigated whether domain-specific factors explained part of the variance in performance (consistent with the view that the mind consists of specialized and at least partially independently evolving modules), or whether and to what extent a general factor accounts for the variance in performance across the different domains. We developed a hierarchical Bayesian model *ad hoc* using data at the individual level because the Bayesian paradigm offers several advantages in this setting: it affords great flexibility in modelling complicated interactions in a hierarchical framework; it readily incorporates latent variables, thereby facilitating comparisons across tasks with different types of responses; and it allows us to handle rank data with ties in a straightforward fashion, best permitting us to perform inference based on the data at our disposal. In contrast, classical analyses would require that we determine the sampling distributions of our statistics (in repeated replications of the entire experimental framework), which is not feasible for these data. If domain-specific factors explained an important part of the variance in performance across domains, we briefly discussed the possible evolutionary pressures that might be linked to different domain-specific cognitive skills in different species. We used data at the level of individual subjects collected with comparable procedures on 19 chimpanzees, 5 bonobos (*Pan paniscus*), 10 orangutans (*Pongo pygmaeus*), 8 gorillas (*Gorilla gorilla*), 18 spider monkeys (*Ateles geoffroyi*), 27 brown capuchin monkeys (*Cebus apella*) and 12 long-tailed macaques. These species differ in phylogenetic relatedness (4 great apes, 2 New and 1 Old World monkeys), and socio-ecological characteristics (e.g. degree of frugivory: lower in gorillas; fission-fusion dynamics: lower in gorillas, capuchin monkeys and macaques; manipulatory skills: higher in *Pan* and capuchin monkeys). If it is true that specific socio-ecological factors are linked to the enhancement of specific cognitive skills (e.g. [23]), then the selected species would enable us to examine the existence of domain-specific factors. The administered tasks assessed cognitive skills across a wide range of domains, including inhibition (e.g. suppressing prepotent responses), memory (e.g. retrieving hidden food after delay), transposition (e.g. keeping track of invisible displacements) and support (e.g. understanding of mean-end connections, by selecting the tool to which food is attached). More than one task was used for each domain to facilitate that only consistent differences across multiple tasks were identified.

## Materials and Methods

### Ethics Statement

Animal husbandry and research comply with the “EAZA Minimum Standards for the Accommodation and Care of Animals in Zoos and Aquaria”, the “WAZA Ethical Guidelines for the Conduct of Research on Animals by Zoos and Aquariums” and the “Guidelines for the Treatment of Animals in Behavioral Research and Teaching” of the Association for the Study of Animal Behavior (ASAB). Subjects were housed in large enclosures and lived in well-established groups with conspecifics. They participated in the tasks on a completely voluntary basis and were never food or water deprived. Data collection consisted in the administration of simple cognitive tasks, which were not invasive and strictly adhered to the legal requirements of Germany, Holland, Mexico and Italy, the countries were the primates were housed. Subjects received extra food when correctly solving the tasks and were never punished for incorrect performance. No medical, toxicological or neurobiological research of any kind was conducted on the subjects tested. All the protocols used in this study were ethically approved by an internal committee at the Max Planck Institute for Evolutionary Anthropology, Germany. Permission to conduct research was also provided by all the other facilities in which the tested primates were housed (the Centenario Zoo in Merida, Mexico for the spider monkeys; the ISTC-CNR Primate Centre in Rome, Italy for the capuchin monkeys; the University of Utrecht, Netherland for the long-tailed macaques; no permission IDs were given). Because our study was purely behavioural/observational, no application to other ethic committees was required.

### Subjects

The study subjects were sexually mature individuals of both sexes and of various ages. Not all subjects were tested in each task, but there were always a combination of sexes and ages (see Table S1). To allow appropriate inter-specific comparisons, all subjects but the spider monkeys had similar experience relevant to the testing situation. Spider monkeys, which had never been tested before in cognitive tasks, went through a longer habituation period to the experimenter and the testing procedures (i.e. being longer trained to enter the testing rooms, to be isolated during testing and to retrieve the food provided from the experimenter out of any experimental context). More information on the animals’ housing conditions and rearing histories are in Supporting Information (S1).

### Administered Tasks

Detailed procedures of the experimental tasks are in Supporting Information (S1) and in [49]–[51]. The basic testing procedure consisted of presenting the subjects with two or more alternatives. Tasks for the inhibition domain entailed subjects (i) refraining from choosing the now empty opaque cup under which they previously retrieved a reward (A not B task, or IN1), (ii) refraining from choosing an empty opaque cup close to an opaque cup from which they previously retrieved a reward (middle cup task, or IN2), (iii) refraining from reaching toward a reward directly through a plexiglas panel and instead taking a detour movement through one hole (plexiglas hole task, or IN3), (iv) refraining from reaching toward a reward directly through a transparent door and instead taking a detour movement through another transparent door to grab the reward from behind (swing door task, or IN4), or (v) refraining from reaching for a smaller immediate reward to obtain a larger delayed one (delay of gratification task, or IN5). For the memory domain, subjects had to retrieve food from under one of three opaque cups after (i) 30 seconds (ME1) or (ii) 30 minutes (ME2). For the transposition domain subjects had to retrieve food under one of three opaque cups after their location had been switched. The transposition tasks consisted in (i) the baited cup switching location with another cup while the third cup remained stationary (TR1), (ii) the baited cup switching location with another cup and then again with the third cup (TR2), (iii) the baited cup switching location with another cup twice, returning to its original location (TR3), or (iv) the unbaited cups switching location while the baited cup remained stationary (TR4). For the support domain subjects had to select between two cloth pieces/strings and pull the one which was attached to the reward. Subjects should select (i) the large cloth piece with a reward on top instead of the large cloth piece with a reward close by (SU1), (ii) the large cloth piece with a reward on top instead of a combination of two small cloth pieces, the accessible of which had no reward on top (SU2), (iii) the large cloth piece under a bridge, with a reward on top of the cloth and under the bridge instead of the large cloth piece under a bridge, with a reward on top of the bridge (SU3), or the long string with a reward on top instead of a combination of two short strings, the accessible of which had no reward on top, with the two strings being (iv) divided by a little gap (SU4), (v) slightly overlapping (SU5) or (vi) adjacent (SU6). The effect of motivation was controlled by using control conditions whenever appropriate [49], [50]. Moreover, no tasks that could bias the results in favour of specific taxa (i.e. requiring enhanced manual or visual skills) were used.

### Assignment of Tasks to the Different Domains

Tasks were assigned to the different domains, according to existing literature (e.g. [25]; also see [49]–[51]). However, we also checked the adequacy of this traditional task assignment using additional discrepancy measures (see the Data analysis section for more details on the statistical procedure). Note that this analysis was aimed at verifying the adequacy of our pre-specified assignment (confirmatory analysis), not at identifying the “optimal” assignment (exploratory analysis), because (i) we did not have an independent data set to evaluate the assignment of tasks to domains, and (ii) we did not want to ignore existing literature when assigning tasks to different domains. The upper bound of the prior-predictive-posterior (PPP) p-value (see [52], [53]) checking if the assignment of the 17 tasks to the 4 domains was inadequate was 0.02, which is consistent with what would be expected from a series of minor misassignments.

For exploratory purposes, we therefore assigned the 17 tasks to 6 different domains. In particular, the inhibition domain was split in two different domains, one including the A not B task (IN1), the middle cup task (IN2) and the delay of gratification task (IN5), and the other one including the plexiglas hole task (IN3) and the swing door task (IN4). Similarly, the memory domain was split in two different domains, one including the 30 seconds memory task (ME1), and one including the 30 minutes memory task (ME2). The rational of this new task assignment was that (i) only in the IN3 and IN4 tasks was plexiglas used in the set-up, and plexiglas is notoriously a confounding factor for several species, the understanding of whose properties might involve cognitive skills of a different domain (e.g. [54]); (ii) the two memory tasks assess short-term and long-term memory, which are thought to belong to two different memory systems (e.g. [55]). This new task assignment was checked for the assignment’s adequacy. The upper bound of the PPP p-value was 0.11, suggesting that although the task assignment might still not be optimal it was good enough given the large quantity of data that were fitted to the model.

Despite these results, we preferred to use the first assignment of tasks to 4 domains because (i) only a much larger independent data set would make us feel comfortable enough to defy the traditional assignment of tasks; (ii) an innovative assignment of tasks would best be done with exploratory factor analysis, which our dataset does not allow, aimed to specifically identify the “optimal” assignment of tasks to domains; (iii) the assignment of tasks to 6 domains was not radically different from the first one, suggesting that although the task assignment we adopted might not have been optimal, no radical changes would anyway be needed to improve the model’s adequacy; and (iv) the 0.02 upper bound of the PPP p-value for the task assignment to 4 domains was still acceptable considering that with the large amount of individual data minor discrepancies might be statistically significant.

### Data Analysi

For the analyses, we used the ratio between the percentage of correct choices in the experimental and in the control trials (for each subject, 1 trial in IN1, 2 trials in IN2), the percentage of correct choices in the experimental trials (2 trials in IN3, TR1 and TR2; 10 trials in IN4; 3 trials in ME1 and ME2; 1 trial in TR3 and TR4; 6 trials in SU1, SU2, SU3, SU4, SU5, SU6) and the indifference pointed reached IN5 (i.e. when the smaller and larger rewards were equally valued) as in Amici and colleagues [49], [50]. The mean value and standard deviation for each species and task are reported in Table S2. The data used in this study have been already published to address specific questions [49]–[51], [56]–[58] with the exception of three tasks in the support domain. For the purpose of this study, we re-analysed them with a hierarchical Bayesian modelling approach, using binomial data for each trial whenever possible (all tasks except IN1, IN2 and IN5) to avoid loss of information (i.e. how an individual performs, instead of simply the rank order of performance at the species level as used in other studies). Our modelling approach allowed us to estimate the amount of variation across the four domains explained by inter-specific differences, which would be interpreted as G.

For the Bayesian modelling, each response on the 17 tasks was assumed to have an underlying latent variable on a continuous scale. This was done to create a more parsimonious model which is amenable to model estimation and comparisons across models and across tasks. The latent variables were modelled with varying degrees of complexity to find a balance between model adequacy and model parsimony. Normally distributed latent performance variables were assumed to determine the observed performance for both the binomial [59] and rank response tasks [60]. In our study, these latent variables were assumed to be affected by up to four random effects: species effects (i.e. G), individual effects, species*domain effects (i.e. domain-specific cognitive modules) and individual*domain effects. These effects were included as random effects, implying that their importance could be assessed by the proportion of the total variance of the latent variables for which they account. In addition, recognizing that none of the models would be expected to perfectly explain subjects’ performance, we included an error term in the latent variable model. The error term was either assumed to have the same variance for each task or allowed to have a task-specific variance hierarchically based on a common error variance.

We formally assessed the adequacy of the various models by computing discrepancy measures (based on [52], [53]), goodness-of-fit quantities that we customized based on the selected model assumption. Model assumptions that were investigated include that no additional random effects were needed, that error variances were the same for all tasks, and that the assignment of tasks to domains was adequate (see above). Each discrepancy measure tends to be larger compared to a reference distribution when the measure’s targeted assumption is violated. The discrepancy measure was compared to a reference distribution to assess the evidence against the model assumption. From the discrepancy measures, we identified an upper bound on the PPP p-value. The actual p-value was exceedingly difficult to obtain, but the upper bound was feasible to compute. For these upper bounds, a value larger than the traditional 0.05 should be used to indicate significance, such as 0.25 [53]. Each candidate model was fitted using a Markov chain Monte Carlo algorithm with at least 50,000 burn-in iterations and then at least 3,000,000 more iterations to obtain precise estimates. More details on the statistical procedures can be found in the Supporting Information (S1) and in Barney [61].

## Results

Table 1 reports the estimated proportion of total variance due to each effect for the various models. The simplest model with no random effects M0 (0,0) was inadequate because the discrepancy measure designed to identify species effects indicated a significant lack of fit. Similarly, we found that the model that included only the species effect M1 (S,0) was inadequate because the discrepancy measure designed to identify species*domain effects gave strong statistical evidence of a species*domain effect. Including species effects and individual effects also resulted in a model M5 (SI,0) with significant lack of fit, due to the omission of species*domain effects. The model with species and species*domain effects M3 (SD,0) did not indicate significant lack of fit stemming from the omission of individual effects. Also, this model (and all other random effects specifications considered) did not indicate a model inadequacy due to the assumption that each task has the same error variance. Indeed, every pair of models (with and without the assumption of constant variance) yielded similar inferences on the relative importance of each effect to explain latent performance.

**Table 1:** Posterior mean (± standard deviation) of variance proportions by source for all models considered. M3 is the best model.

MODEL	Error: σ^2^± sd	Species: σ^2^± sd	Species* domain:σ^2^± sd	Individual:σ^2^± sd	Individual* domain:σ^2^± sd
**M0 (0,0)**	1.00				
**M1 (S,0)**	0.87±0.07	0.13±0.07			
**M2 (S,J)**	0.86±0.08	0.14±0.08			
**M3 (SD,0)**	0.66±0.08	0.17±0.09	0.17±0.06		
**M4 (SD,J)**	0.67±0.08	0.16±0.09	0.16±0.06		
**M5 (SI,0)**	0.87±0.07	0.12±0.07		0.01±0.01	
**M6 (SI,J)**	0.85±0.07	0.14±0.07		0.02±0.01	
**M7 (SDID,0)**	0.67±0.07	0.15±0.08	0.16±0.05	0.01±0.01	0.02±0.01
**M8 (SDID,J)**	0.67±0.07	0.14±0.08	0.16±0.05	0.01±0.01	0.03±0.01

M0, M1, M3, M5 and M7 assume that the error variance is identical across tasks (0), while M2, M4, M6 and M8 allow the error variance to have a task-specific variance hierarchically based on a common error variance (J). M0 includes no random effects. M1 and M2 only include the species effect (S). M3 and M4 include species and species*domain effects (SD). M5 and M6 include species and individual effects (SI). M7 and M8 include species, species*domain, individual and individual*domain effects (SDID). Models M0–M2 and M5–M6 had significant lack of fit because of their failure to include both species and species*domain effects; this is also reflected in the sizable proportion of variance that both the species and species*domain effects account for whenever they were included in these models (i.e. M3–M4 and M7–M8). The principal conclusions drawn from M3 are similar to those from M4, M7, and M8. Because the discrepancy measures did not suggest that M3 needed individual effects (as in M7 and M8) or that the error variances needed to deviate per task (as in M4), M3 was the best model (see [60] for a formal assessment approach).

The best model was therefore M3 (SD,0), with species and species*domain effects and a common error variance for the latent variables of all tasks (Table 1). In the best model, both the species and the species*domain effect were estimated to explain 17% of the variance, with the error term accounting for 66% of the variance. Because different experimenters tested different species, the species*domain effect could be influenced by the confounding effect of different experimenters. In order to control for this possible confounding effect, we rerun the analyses only including the 3 monkey species tested by the same experimenter (the first author) and found that the amount of variance explained by species*domain effects (13%) was similar to that of the original analysis based on the seven species.

The simpler models were inadequate because they excluded either species or species*domain effects, both of which were necessary based on the discrepancy measures and the considerable proportion of the variance attributed to each. The more complex model with species, species*domain, individual and individual*domain effects M7 (SDID,0) did not seem necessary (i) because the discrepancy measure did not even detect individual effects should have been added to M3 (SD,0), and (ii) because the individual and individual*domain effects were estimated to account together for less than 4% of the total variance (M5–M8 in Table 1).

**Figure 1:**
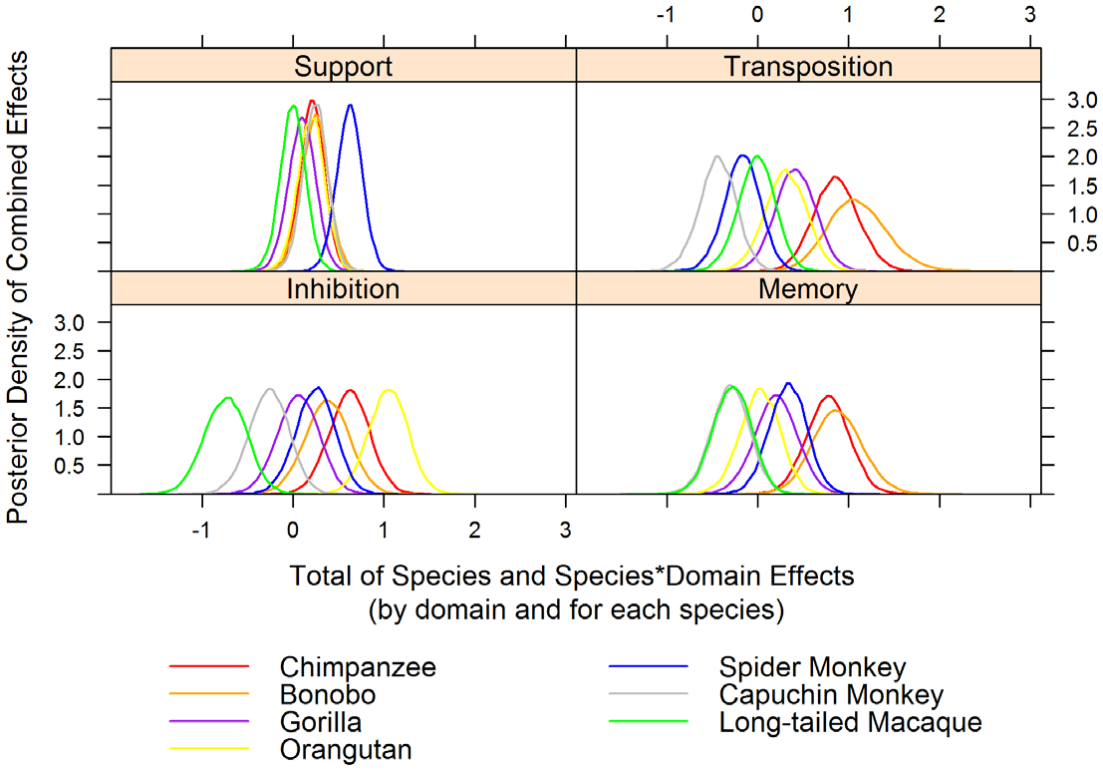
The estimated marginal posterior distributions of the combined species and species*domain effects for each species in each domain. They convey the uncertainty in the combined effects of both domain-general and domain-specific factors. Larger average values of a latent variable increase the likelihood of good performance, and narrower curves reflect greater precision in identifying the combined effects.

**Figure 2:**
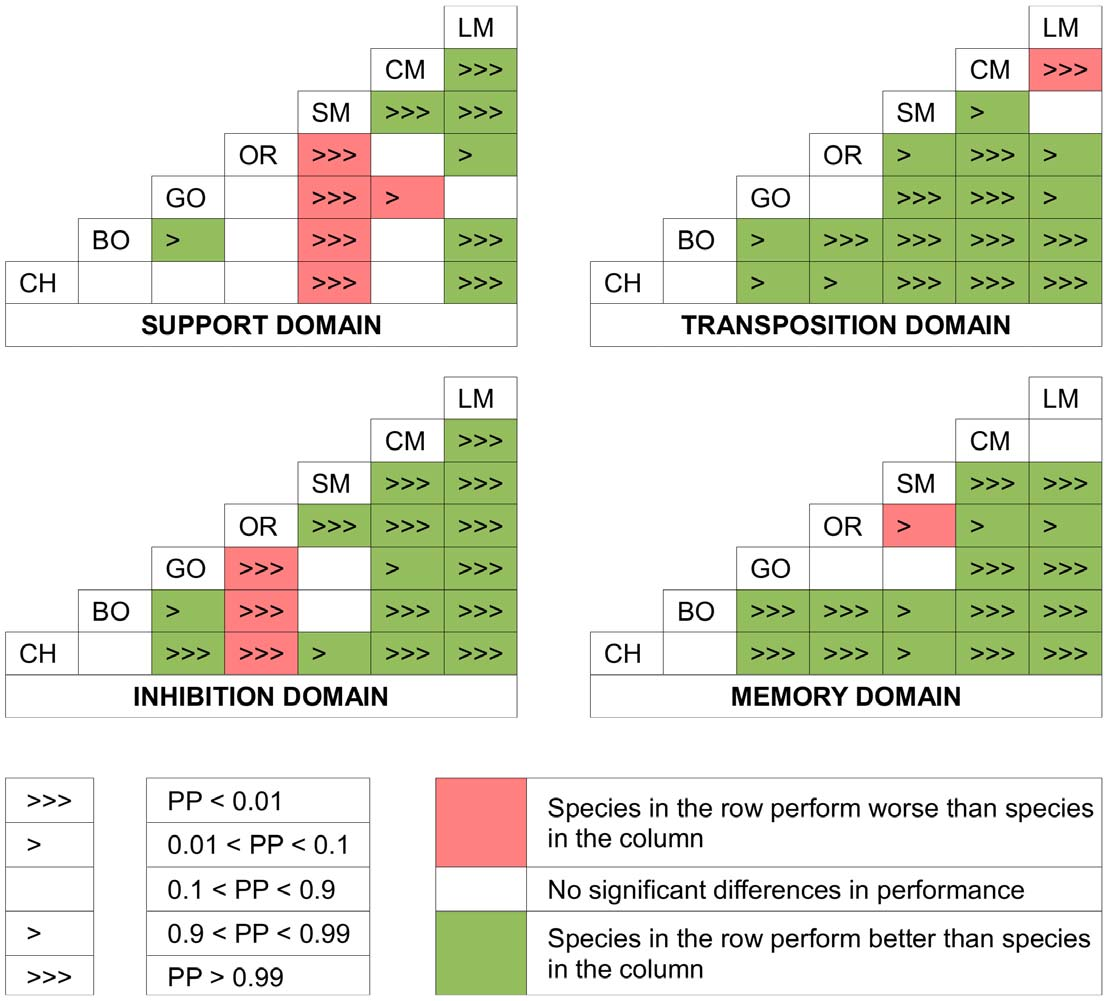
For each domain, image plots of the posterior probability (PP) that the listed row species performed better than the listed column species on average (CH = chimpanzees, BO = bonobos, GO = gorilla, OR = orangutans, SM = spider monkeys, CM = capuchin monkeys, LM = long-tailed macaques). Values close to 0 (pink shade) indicate the row species perform worse than the column species, whereas values close to 1 (green shade) indicate the row species perform better than the column species. The plots more directly reflect the evidence for differences between species in the combined effects.

The combined species and species*domain effects do not display the same pattern of inter-specific differences for each domain (Figures 1 and 2; only posterior probabilities >0.99 are reported in the text below). In the support domain, spider monkeys’ performance was better relative to that of all other species than expected based on the performance in the other domains, and *Pan* species and capuchin monkeys performed better than macaques. In the transposition domain, *Pan* species’ performance was better relative to that of the three monkey species than expected based on the performance in the other domains. Bonobos performed better than orangutans, orangutans and macaques better than capuchin monkeys, and gorillas better than spider and capuchin monkeys. In the inhibition domain, orangutans’ performance was better relative to that of all other species than expected based on the performance in the other domains, and all other species performed better than macaques. In addition, chimpanzees performed better than gorillas, and the two *Pan* species and spider monkeys performed better than capuchin monkeys. In the memory domain, *Pan* species performance was better relative to that of gorillas, orangutans, capuchin monkeys and macaques than expected based on the performance in the other domains. Spider monkeys and gorillas performed better than capuchin monkeys and macaques.

To assess whether other studies may have failed to detect species*domain effects simply due to the way data were used, we also modelled ranks of species-level means (instead of rank data and binomial data at the trial level for each individual). The results of these analyses are consistent with our previous results: the best model included species effects and species*domain effects, with each effect accounting for an estimated 24% and 32% of the total variance in the latent variables, respectively. In this case, the species and species*domain effects appear to be more prominent, probably because the variance attributable to what is left over in the model (“pure error”) is relatively less influential for averages than for individual data.

## Discussion

In this study, both the species and the species*domain effect were estimated to explain 17% of the variance in performance. The same pattern of results was obtained when analysing rank data at the species level as in other studies. The error term accounted for 66% of the observed variance, whereas individual and individual*domain effects needed not be included to explain the observed variance. These results support the idea that a single general factor cannot alone explain the variance in performance across different domains, and that the mind of primates is (at least partially) modular, with domain-specific cognitive skills undergoing different evolutionary pressures in different species in response to specific ecological and social demands.

Some species performed better than others in some, but not all, domains. In the support domain, for example, spider monkeys performed much better than the other six primates, which is consistent with Harlow & Settlage’s [62] findings based on the comparison of nine primate species. The spider monkeys’ impressive performance has been explained in terms of their relative high degree of fission–fusion dynamics [50]. A high degree of fission-fusion dynamics occurs when group members often split and merge in subgroups of flexible membership [63]. This results in group members potentially being apart for extended periods and therefore dealing with especially fragmented social information. Such individuals would have then to retain much “off-line” information and would especially benefit from an enhanced ability to better understand relations between relations (i.e. analogical reasoning) and reduce the cognitive load [39], [63]. In the support domain, the enhancement of these abilities could result in species with higher levels of fission–fusion dynamics to better understand the physical relationship between a functional tool and the food in one task, and extend this knowledge to other tasks [50].

Orangutans performed especially well in the inhibition domain, which is consistent with orangutans having a relatively large orbital frontal cortex, a brain area associated with inhibitory skills [64], [65]. Orangutans’ performance in inhibitory tasks has already been linked to their extremely dispersed social system which reduces direct individual food competition and might be linked to orangutans being less impulsive and thus better able to assess situations before acting [49], [58]. In addition, bonobos and chimpanzees performed similarly across all domains, and great apes did not overall outperform the three monkey species (similarly, see [40]).

The importance of domain-specific factors in our study appears to contrast with Deaner and colleagues’ [26] lack of evidence of an interaction between genus and domain. Several reasons may account for this difference. First, our study assessed primate cognitive performance using data at the individual level for each task, whereas Deaner and colleagues [26] used data from different studies and thus had to rank data at the genus level for modelling. One could therefore argue that our study relied on more precise information. This explanation, however, was not supported by us re-running the analyses with models using ranks of species level means as the results did not substantially change. Second, our study included fewer species than Deaner and colleagues [26], and crucially, their performance was distributed more narrowly than those included in Deaner and colleagues’ [26] study. In fact, our study included species whose overall cognitive performance was among the best according to Deaner and colleagues’ [26] comparison across 24 primate genera. Although a narrower range in performance may have contributed to reduce the likelihood of finding a G factor, it is also possible that the greater species homogeneity in our sample could have had the opposite effect, and contributed it to promote the appearance of G. Therefore a narrower range, either in terms of species or their performance, does not seem a satisfactory explanation for the difference between the studies. Third, the studies may have produced different results due to differences in the domains included. Deaner and colleagues [26], for example, did not include tasks in the inhibition domain (with the only exception of the reversal learning task, the ability of which in measuring inhibitory skills is however controversial: [66], [67]), but they did include tool use and object-discrimination learning domains, in which great apes notoriously perform better than other species (e.g. [25]). Moreover, our tasks had been especially designed to allow inter-specific comparisons, so that the tasks were as basic as possible and subjects were not required to understand complex contingencies of the tasks, reducing the role played by other cognitive skills when assessing how subjects performed on the selected cognitive skills. Fourth, in our study the seven species were tested on a similar range of domains, whereas Deaner and colleagues [26] had to rely on published data, which were not evenly distributed across species. As a consequence, domain-specific effects might have been more easily detected in our study because more data were available on all tasks that were tested across all the species. In this respect, selecting basic tasks that address an array of cognitive skills belonging to a wide range of domains and systematically administering them to all study species might allow the detection of domain-specific effects that would otherwise be “masked”. Great apes, for example, do indeed perform better than most monkeys in most domains, but not in all domains. The lack of data on a wide range of domains for each species may conceal the role played by domain-specific factors.

Based on our analyses, the species effect accounts for an estimated 17% of the variance in latent performance across different cognitive domains. One explanation for the co-existence of the species effect with the domain-specific effect is that even if specific cognitive processes are localized in discrete brain regions, reflecting the taxon’s specific adaptations to particular ecological problems, some properties of the brain, such as the amount of grey matter, are intercorrelated across brain regions, possibly affecting all cognitive domains [1], [28]–[31], [68]. For example, Lee [28] proposed that more synaptic connections might enhance the overall processing power of the brain, regardless of the brain regions involved. This is not in contrast with the view that specific cognitive processes are localized in discrete brain regions and reflect responses to specific ecological and social demands, as having more synaptic connections and specific cognitive processes in discrete brain regions are two different characteristics of the brain, which are not mutually exclusive. Moreover, it is conceivable that some basic cognitive skills (such as memory and object permanence) are at play in all the domains (see e.g. [69] for a review on the relationship between working memory and G). Indeed, it seems hard to conceive single cognitive domains in which *only* one cognitive skill is required. The species effect we found in our study might thus be due to some (independently evolved) cognitive skills being usually needed in all tasks and domains, like working memory. At the moment, our data do not allow us to understand to what extent the species effect is a pure indicator of G. This is because, although we have selected tasks which are widely considered to require specific cognitive skills, we cannot rule out that the tasks required partially overlapping cognitive skills belonging to different domains. Still, we showed the presence of domain-specific factors and that the co-existence of domain-specific and domain-general factors is possible, as other studies also suggest (e.g. [8]).

In our study, the error term accounted for 66% of the observed variance. This is not surprising if we consider that performance in cognitive tasks is only a crude representation of cognitive skills, introducing an important loss of information and enhancing the contribution of error in our model. Moreover, the contribution of the error is of course more apparent for individual-level observations (like those used in our study) than for species-level averages (like, for example, those used by Deaner and colleagues [26]).

In our study, inter-individual differences within species played a minimal role. When we added individual and individual*domain effects to the model, they were estimated to explain together less than 4% of the total variance in latent performance. This suggests that there was substantially more systematic inter-specific variation than systematic intra-specific variation in terms of cognitive performance in the seven tested primate species, with inter-individual differences possibly playing a more limited role than in humans (e.g. [68]). One consequence of this result is that the taxonomic differences detected in our study are not merely the consequence of measurements collected from a few exceptional individuals (cf. [26]). It is however possible that studies including more individuals will find larger inter-individual variation than reported here.

Because multiple researchers collected the data, the results could have been affected by an experimenter effect. Indeed, there was a high degree of confounding between the experimenter and the species and species*domain effects. For example, all assessments from the three monkey species were conducted by the same experimenter, and four of the six experimenters collected data from only one domain. Therefore, the confounding between the experimenter effect and the species and species*domain effects was too severe to be reliably assessed directly. When we rerun the analyses only including the 3 monkey species, so controlling for the possible confounding experimenter effect, we found that similar results to those of the analysis based on the seven species. Thus, our results seem solid, although we could not fully control for the experimenter effect.

In conclusion, our results provide evidence for the existence of a modular mind in primates, with possibly several specialized modules having independently evolved as a response to different selective pressures [15], [23]–[25], [40]. Interestingly, our results might also explain why, despite the importance of domain-specific effects, great apes have often outperformed monkeys. Great apes might indeed perform better than most monkeys in most (but not all) domains, and using complex tasks requiring cognitive skills from multiple domains might more probably involve also one of those skills which are especially enhanced in great apes, but not in other species, preventing the detection of previously unreported domain-specific effects. It is therefore desirable that researchers coordinate their efforts to agree on simple standardized methods to test a wide range of cognitive skills from different domains [41], in order to better address the question of modularity through the use of a large data-set including numerous species differing in socio-ecological characteristics, phylogenetic relatedness and other relevant characteristics.

## Supporting Information

Table S1 **Characteristics of each tested subject and the administered tasks and domains.**
(DOCX)

Table S2 **For each of the tested species, mean performance (± SD) in each task.**
(DOCX)

Supporting Information S1 **Housing conditions and rearing histories of tested subjects; detailed experimental procedures; modelling details; references.**
(DOCX)
