# Supplementary material for: A Modular Mind? A Test Using Individual Data from Seven Primate Species
Source: PLoS One. 2012 Dec 19;7(12):e51918. doi: 10.1371/journal.pone.0051918 (PMC3526483; doi:10.1371/journal.pone.0051918)
Supplement: Table S1 — Characteristics of each tested subject and the administered tasks and domains. (DOCX) [file pone.0051918.s001.docx]

**TABLE S1**

Characteristics of each tested subject (species, sex and age) and the administered tasks and domains.

| *Subject* | *Species* | *Sex* | *Age* | *Domains and tasks* |
| --- | --- | --- | --- | --- |
| 1 | Spider monkey | F | S | All but IN3 |
| 2 |  | F | S | All |
| 3 |  | M | S | All |
| 4 |  | M | S | All |
| 8 |  | F | A | All |
| 9 |  | F | A | All |
| 11 |  | F | A | All but IN3 |
| 21 |  | M | A | All but IN3 |
| 22 |  | M | A | All but IN3 and IN5 |
| 23 |  | M | A | All but IN3 |
| 24 |  | F | S | All |
| 26 |  | F | A | IN1-2, TR1-4 |
| 31 |  | F | A | All but IN3 |
| 32 |  | F | A | IN2, IN5, ME1-2 |
| 34 |  | M | A | IN2, ME1-2 |
| 41 |  | M | A | IN2 |
| 42 |  | F | S | IN1-2 |
| 43 |  | F | A | IN1 |
| Brahms | Capuchin monkey | F | A | IN1-3 |
| Cognac |  | M | A | All but IN3 |
| Narciso |  | M | A | IN3 |
| Pandora |  | F | A | IN1-2 |
| Panna |  | F | A | IN1-2 |
| Paquita |  | F | A | All but IN1-3 |
| Patè |  | M | A | IN1-3 |
| Pedro |  | M | S | All but IN3 |
| Penelope |  | F | S | All but IN3 |
| Pepe |  | M | A | All |
| Pippi |  | F | A | All |
| Punk |  | F | A | IN1-2 |
| Quincey |  | F | J | All but IN1-3 |
| Roberta |  | F | A | All but IN1-2 |
| Robin Hood |  | M | A | All |
| Robinia |  | F | A | IN1-3 |
| Robiola |  | F | A | IN3 |
| Robot |  | M | A | IN1-3 |
| Rosso |  | M | A | IN1-2 |
| Rubens |  | M | S | All but IN1-3 |
| Rucola |  | F | S | IN1-3 |
| Sandokan |  | M | S | All but IN1-3 |
| Saroma |  | F | S | IN1-3 |
| Viola |  | M | A | IN1-3 |
| Virginia |  | F | S | IN3 |
| Vispo |  | M | S | All |
| Zapotec |  | M | A | IN1-3 |
| Anastasia | Long-tailed macaque | F | J | All |
| Cleo |  | M | A | All |
| Cornea |  | F | A | All |
| Era |  | F | A | All |
| Icetea |  | F | A | All |
| Linea |  | F | S | All |
| Logica |  | F | S | All |
| Ofelia |  | F | J | All |
| Salvadoro |  | M | J | All |
| Sea |  | F | A | All |
| Video |  | M | S | All |
| Zargasso |  | M | J | All |
| Alex | Chimpanzee | M | S | SU1-6 |
| Alexandra |  | F | S | SU1-6 |
| Annett |  | F | S | SU1-6 |
| Brent |  | M | S | SU1-6 |
| Corry |  | F | A | ME1-2, SU1-6 |
| Dorien |  | F | A | IN3, IN5, ME1-2, SU1-6 |
| Fifi |  | F | A | All but IN5 |
| Fraukje |  | F | A | All |
| Frodo |  | M | A | All but IN5 |
| Jahaga |  | F | A | All but IN5 |
| Lomè |  | M | S | ME1-2 |
| Natascha |  | F | A | SU1-6 |
| Patrick |  | M | S | IN5, SU1-6 |
| Pia |  | F | S | SU1-6 |
| Riet |  | F | A | All but IN5 |
| Robert |  | M | A | IN5, ME1-2, SU1-6 |
| Sandra |  | F | A | All but IN4 |
| Trudi |  | F | A | All but IN5 |
| Ulla |  | F | A | SU1-6 |
| Joey | Bonobo | M | A | All |
| Kuno |  | M | A | All |
| Limbuko |  | M | A | All |
| Ulindi |  | F | A | All |
| Yasa |  | F | A | IN5, ME1-2, SU1-6 |
| Bimbo | Orangutan | M | A | All |
| Dokana |  | F | A | IN4-5, ME1-2, SU1-6 |
| Dunja |  | F | A | All |
| Kila |  | F | S | IN5, ME1-2 |
| Padana |  | F | A | All |
| Pagai |  | M | J | IN5, ME1-2 |
| Pini |  | F | A | All |
| Raja |  | F | J | IN5, ME1-2 |
| Toba |  | F | A | IN1-4, ME1-2, TR1-4 |
| Walter |  | M | A | IN1-4, ME1-2, TR1-4 |
| Bebe | Gorilla | F | A | All |
| Gorgo |  | M | A | All |
| Kibara |  | F | J | IN5, ME1-2 |
| N’diki |  | F | A | All but IN5 |
| N’kwango |  | M | A | IN1-4, ME1-2, TR1-4 |
| Ruby |  | F | A | All but IN5 |
| Viringika |  | F | A | All |
| Vizuri |  | M | A | IN1-2, ME1-2, TR1-4 |

M=male, F=female; A=adult, S=subadult, J=juvenile (according to [1,2]); IN=inhibition domains, ME=memory domains, TR=transposition domains, SU=support domains. See the text for more details on each task, as identified by the numbers (e.g. IN1).
