# Supplementary material for: A Modular Mind? A Test Using Individual Data from Seven Primate Species
Source: PLoS One. 2012 Dec 19;7(12):e51918. doi: 10.1371/journal.pone.0051918 (PMC3526483; doi:10.1371/journal.pone.0051918)
Supplement: Table S2 — For each of the tested species, mean performance (± SD) in each task. (DOCX) [file pone.0051918.s002.docx]

**TABLE S2**

For each of the tested species, mean performance (± SD) in each task.

|  | INHIBITION  DOMAIN | | | | | MEMORY  DOMAIN | | TRANPOSITION  DOMAIN | | | | SUPPORT  DOMAIN | | | | | |
| --- | --- | --- | --- | --- | --- | --- | --- | --- | --- | --- | --- | --- | --- | --- | --- | --- | --- |
| SPECIES | AnotB | Mid  Cup | Ple Hol | Swi Doo | Del Gra | 30  ss | 30 min | TR1 | TR2 | TR3 | TR4 | SU1 (Cl) | SU2 (Cl) | SU3 (Cl) | SU4 (St) | SU5 (St) | SU6 (St) |
| Chimpanzees | 100.0 ± 0.0 | 57.1 ± 45.0 | 93.8 ± 17.7 | 6.7 ± 16.3 | 122.6± 35.5 | 93.9 ± 13.5 | 85.7 ± 17.8 | 100.0± 0.0 | 78.6 ± 26.7 | 100.0± 0.0 | 100.0± 0.0 | 71.3 ± 17.0 | 63.9 ± 15.4 | 67.6 ± 19.4 | 64.7 ± 24.2 | 52.9 ± 20.6 | 52.9 ± 16.9 |
| Bonobos | 100.0 ± 0.0 | 75.0 ± 28.9 | 87.5 ± 25.0 | 0.0 | 74.4 ± 19.0 | 100.0 ± 0.0 | 86.7 ± 29.8 | 100.0 ± 0.0 | 100.0 ± 0.0 | 100.0 ± 0.0 | 100.0 ± 0.0 | 66.7 ± 23.6 | 50.0  ± 11.8 | 73.3  ± 22.4 | 63.3  ± 21.7 | 70.0  ± 27.4 | 56.7  ± 25.3 |
| Gorillas | 100.0 ± 0.0 | 21.4 ± 39.3 | 41.7 ± 37.6 | 8.3 ± 20.4 | 44.0 ± 21.7 | 83.4 ± 17.8 | 50.0 ± 19.3 | 78.6 ± 26.7 | 78.6 ± 26.7 | 71.4 ± 48.8 | 100.0 ± 0.0 | 66.7  ± 11.8 | 63.4  ± 7.5 | 40.0  ± 25.3 | 66.7  ± 16.7 | 53.3  ± 18.3 | 53.3  ± 27.4 |
| Orangutans | 83.3 ± 40.8 | 66.7 ± 51.6 | 100.0 ± 0.0 | 38.6  ± 32.4 | 49.6  ± 25.0 | 66.6  ± 27.4 | 54.2  ± 35.4 | 66.7  ± 25.8 | 66.7  ± 40.8 | 83.3  ± 40.8 | 100.0 ± 0.0 | 73.3  ± 9.1 | 46.7  ± 14.0 | 80.0  ± 13.9 | 66.7  ± 16.7 | 46.7  ± 14.0 | 56.7  ± 19.0 |
| Spider monkeys | 93.3  ± 25.8 | 79.4  ± 25.4 | 66.7  ± 40.8 | 0.0 | 76.0  ± 27.9 | 88.1  ± 16.6 | 61.9  ± 28.8 | 53.8  ± 24.7 | 38.5  ± 36.3 | 53.8  ± 51.9 | 84.6  ± 37.6 | 84.7  ± 18.1 | 68.1  ± 25.1 | 83.3  ± 18.8 | 90.3  ± 13.2 | 70.8  ± 17.6 | 80.6  ± 13.9 |
| Capuchin monkeys | 78.9  ± 41.9 | 34.2  ± 41.0 | 78.1  ± 31.5 | 0.0 | 21.7  ± 9.0 | 47.2  ± 26.5 | 44.4  ± 26.0 | 25.0  ± 33.7 | 33.3  ± 32.6 | 50.0  ± 52.2 | 83.3  ± 38.9 | 59.7  ± 18.1 | 51.4  ± 20.7 | 65.3  ± 21.8 | 69.4  ± 21.1 | 63.9  ± 23.4 | 76.4  ± 18.1 |
| Long-tailed macaques | 66.7  ± 49.2 | 45.8  ±49.8 | 4.2  ±14.4 | 0.0 | 24.3  ± 12.8 | 52.8  ± 26.5 | 41.7  ± 28.9 | 66.7  ± 24.6 | 62.5  ± 31.1 | 50.0  ± 52.2 | 75.0  ± 45.2 | 59.7  ± 13.2 | 55.6  ± 13.0 | 59.7  ± 15.0 | 41.7  ± 24.1 | 52.8  ± 15.6 | 45.8  ± 21.5 |

In the A-not-B task (AnotB) and in the Middle cup task (Mid Cup), performance is the percentage of correct choices in the first/ first two experimental trials on the percentage of correct choices in the first/ first two control trials; in the Plexiglas hole task (Ple Hol) and in the Swing door task (Swi Doo), performance is the percentage of correct choices in the first two/ first ten experimental trials; in the Delay of gratification task (Del Gra), it is the indifference point reached (in seconds). In the two Memory tasks (30ss and 30 min), performance is the percentage of correct choices in the first three experimental trials. In two Transposition tasks (TR1 and TR2), performance is the percentage of correct choices in the first two experimental trials; in the other two Transposition tasks (TR3 and TR4), it is the percentage of correct choices in the first experimental trial. In the six Support tasks (SU1, SU2, SU3 – all with cloth pieces: Cl – and SU4, SU5, SU6 – all with strings: St), performance is the percentage of correct choices in the first six experimental trials.
