# Supplementary material for: A Modular Mind? A Test Using Individual Data from Seven Primate Species
Source: PLoS One. 2012 Dec 19;7(12):e51918. doi: 10.1371/journal.pone.0051918 (PMC3526483; doi:10.1371/journal.pone.0051918)
Supplement: Supporting Information S1 — Housing conditions and rearing histories of tested subjects; detailed experimental procedures; modelling details; references. (DOCX) [file pone.0051918.s003.docx]

**HOUSING CONDITIONS AND REARING HISTORY OF TESTED SUBJECTS**

Housing conditions.

All subjects were housed in enclosures with outdoor and indoor areas. Outdoor areas were enriched with trees, bushes, climbing structures, ropes, water pools, nets or ropes. Before and during this study, all subjects were fed according to their usual schedule. All subjects were used to being isolated in the section of their enclosures that was used as the testing room (capuchin monkeys: 2 x 2.3 x 2.8 m; long-tailed macaques: 3 x 3 x 2 m; spider monkeys: an isosceles triangular base with 3 m-hypotenuse and 1.5 m height, 3 m high; great apes: an irregular area of circa 9 m^2^, 3 m high). Prior to the testing, all subjects were habituated to the experimenter’s presence.

Rearing histories.

All subjects were born and reared in captivity, except for one gorilla and the spider monkeys who were born in the wild and reared in captivity. Spider monkeys were raised as pets before being rescued, and had extensive experience in the captive environment. Details of their previous experience as pets are not known, as the people who rescued them were not the same as those working in the facilities in which the spider monkeys were housed. Therefore, it is not possible to evaluate how their previous experience as pets might have affected their cognitive performance in the tasks used in this study.

All subjects lived with conspecifics of both sexes and of various ages.

**DETAILED EXPERIMENTAL PROCEDURES**

The inhibition domain.

IN1 - A not B task: refraining from choosing the now empty opaque cup under which a food reward has been previously retrieves. Three identical opaque plastic cups are aligned on a rigid platform between the subject (S) and the experimenter (E), one at the E’s left side, one at E’s right side and one in the middle. On the beginning of each trial, E opens all the three cups to show S that they are empty by letting them rest on one side. Then, in full view of S, E baits one of the two external cups (e.g. the left cup) and then closes all the cups by putting them upside down and occluding the reward. The platform is then pushed towards S, so that S can choose one of the three cups by touching it, pointing to it or trying to reach for it (control trial). The same cup is baited and the procedure repeated until S has made the correct choice for three consecutive trials. Then the same external cup is baited again. After all the cups have been closed, in complete view of S, E opens the baited cup, retrieves the reward, closes the cup again and places the reward under the opposite external cup (e.g. the right cup; experimental trial). E scores whether S chooses the baited location in the control trial and in the experimental trial. The side of the baited cup in the first trial is randomly selected across Ss.

IN2 - Middle cup task: refraining from choosing an empty opaque cup close to an opaque cup from which a food reward has been previously retrieved. Three identical opaque plastic cups are aligned on a rigid platform between S and E, one at the E’s left side, one at E’s right side and one in the middle. On the beginning of each trial, E opens all the three cups to show S that they are empty by letting them rest on one side. Then, in full view of S, E places two rewards in front of two of the three cups (one below each of the two) and then closes all the cups by putting them upside down and occluding the reward. The platform is then pushed towards S, so that S can choose two of the three cups by touching them, pointing to them or trying to reach for them. In the control condition, E baits two cups next to each other. In the experimental condition, E baits the two outer cups, thereby leaving the middle cup unbaited. E scores whether S chooses the two baited cups in both conditions. These two conditions are alternated in a randomized order and the first administered condition is randomly selected across Ss.

IN3 - Plexiglas hole task: refraining from reaching toward a food reward directly through a plexiglas panel and instead take a detour movement through one hole. We use a plexiglas panel about 65 cm wide and 50 high, with one hole at its right bottom side and one hole at its left bottom side, both wide enough to allow S to stick an arm through them. The panel is hanging between E and S, standing upright on the ground and fixed in such a way that S can only reach for the reward placed on the rigid platform on the E’s side by sticking one arm through the holes in the panel. E places one reward directly on the platform, in front of one of the two holes. Then E pushes the platform toward S and lets S retrieve the reward by grabbing it. E repeats the procedure five times more, putting the reward three times on the right side and three times on the left one in a randomized fashion. On the seventh trial, E places the reward on the rigid platform between the two holes. Then E pushes the platform toward S and lets S retrieve the raisin. E scores whether S takes a detour movement through one of the holes in the seventh trial, instead of attempting to reach for the reward directly through the plexiglas panel.

IN4 - Swing door task: refraining from reaching toward a food reward directly through a non-baited door and instead grabbing it from behind. We use a completely transparent box made of plexiglas, with two little doors only opening toward the inside and two little shelves behind the doors where a reward can be placed. The size of each door easily allows S to enter the arm through it, to reach for the reward placed behind the other door, and the box is placed outside S’s enclosure but within S’s reach. The doors only open towards the inside, so that if S tries to reach for the reward by pushing the door directly in front of the reward, the reward falls and S can’t get it anymore. No previous training is needed. Without allowing S to see the baiting procedure, E places one reward behind one of the two doors. E scores whether S takes a detour movement through the unbaited door, instead of attempting to reach for the reward directly through the baited door. The location of the reward is alternated in a randomized order and the first baited side is randomly selected across Ss.

IN5 - Delay of gratification task: refraining from reaching for a smaller immediate food reward to obtain a larger delayed one. On the beginning of each session, E administers four forced-choice trials by presenting S with only one reward quantity (either 1or 3 rewards) and then ten free-choice trials by presenting S with two reward quantities (1 and 3 rewards). The food is presented either on the platform (if subjects had successfully retrieved it in 3 consecutive trials administered prior to this task), or on E’s hands. In both cases, if S chooses the 1 reward by touching it, pointing to it or trying to reach for it, E lets S retrieve it immediately; if S chooses 3 rewards, however, S has to wait a certain delay of time in order to retrieve the reward (see below). After S has made a choice, E removes the non-chosen reward and lets S retrieve the reward; if there is a delay period, E sits looking at the ground and after the delay is over E pushes the chosen reward forward so that S can access the reward. When S has placed the last piece of reward in its mouth, E waits 30 seconds before starting the next trial. 1 reward is always immediately available (~0 ss), while the 3 rewards are immediately available in the first two sessions and then adjusted based on S's performance. The delay for the 3 rewards is incremented past 0 ss only after S has chosen them at least 9 times in the 10 free-choice trials. After these two consecutive successful trials, the following increments are used: choosing 3 rewards 8-10 times increases the delay of the following session of 10 ss; choosing them 6-7 times increases the delay of 5 ss; choosing them 5 times leads to no variation; choosing them 3-4 times decreases the delay of 5 ss; choosing them 1-2 times decreases the delay of 10 ss. If S chooses the same side at least eight times on the free choice trials S is considered to have a side bias, so the session is concluded and repeated on the next day. If S completes 4 consecutive sessions with bias on the same side, prior to the next session S receives 6 forced trials on the side it is not picking (for example, a right-biased S receives 2L, 6L, 2L, 6L…etc., and then the actual session as usual). E scores the reward chosen by S in each trial and calculates the indifference point (the point at which subjects equally valued the smaller immediate reward and the larger delayed reward), obtained when the mean delay for the larger reward of the last five completed sessions does not differ from the mean delay of the preceding five sessions by more than 10%, and all the delays in these last 5 sessions are within a 30 ss range. The mean delay of the last five sessions is the estimation of S's indifference point. The side of the larger reward is randomly assigned and counterbalanced within each session, not repeating the same location more than three times in a row.

The memory domain.

ME1: retrieving a food reward from under one of three opaque cups after 30 seconds. Three identical opaque plastic cups are aligned on a platform between S and E, one at the E’s left side, one at E’s right side and one in the middle. On the beginning of each trial, E opens all the three cups to show S that they are empty by letting them rest on one side. Then, in full view of S, E baits one of the three cups and then closes all the cups by putting them upside down and occluding the reward. After 30 ss, the platform is pushed toward S, so that S can choose one of the three cups by touching it, pointing to it or trying to reach for it. E scores whether S chooses the baited location in each trial. The location of the reward is randomly assigned to the left, middle and right cup and counterbalanced within each S, with the location of the first reward being randomly assigned across Ss.

ME2: retrieving a food reward from under one of three opaque cups after 30 minutes. As before, but 30 minutes elapse between the baiting procedure and the moment in which S can choose a cup.

The transposition domain.

TR1: retrieving a food reward under one of three opaque cups after the baited cup has switched location with another cup while the third cup remains stationary. Three identical opaque plastic cups are aligned on a platform between S and E, one at the E’s left side, one at E’s right side and one in the middle. On the beginning of each trial, E opens all the three cups to show S that they are empty by letting them rest on one side. Then, in full view of S, E baits one of the three cups, closes all the cups by putting them upside down and occluding the reward and performs a transposition, so that the baited cup and another either adjacent or non-adjacent empty cup switch location. E scores whether S chooses the baited cup, by touching it, pointing to it or trying to reach for it. The location of the baited cup is randomly assigned to the left, middle and right cup, with the location of the first reward being randomly assigned across Ss.

TR2: retrieving a food reward under one of three opaque cups after the baited cup has switched location with another cup and then again with the third cup. As before, but during the transposition the baited cup switches location with the next adjacent cup thereby moving to the outer location, or it switches location with the non-adjacent cup and then with the adjacent cup thereby returning to the middle location.

TR3: retrieving a food reward under one of three opaque cups after the baited cup has switched location with another cup twice, returning to its original location. As before, but during the transposition the baited cup and another cup switch locations twice, the reward returning to its initial location.

TR4: retrieving a food reward under one of three opaque cups after the unbaited cups have switched location while the baited cup remains stationary. As before, but during the transposition only the two unbaited cups switch location once.

The support domain.

SU1: selecting between two cloth pieces and pulling the cloth piece with a food reward on top, instead of the cloth piece with a reward close by. Without allowing S to see the baiting procedure, E places two identical cloth pieces (15 cm × 20 cm) on the two sides of a platform positioned between E and S. One reward is placed at the far end of one cloth piece, and another one is placed on the platform, directly next to the far end of the other cloth piece. Both rewards are far enough from S to prevent S from directly retrieving them. Then, E pushes the platform toward S, who can only obtain the reward by pulling the cloth with the reward on top of it. E scores whether S successfully retrieves the reward by pulling the cloth with the reward on its top. The position of the accessible reward is counterbalanced, appearing an equal number of times on both sides and not on the same side for more than two trials in a row.

SU2: selecting between two cloth pieces and pulling the large cloth piece with a food reward on top, instead of a combination of two small cloth pieces, the accessible of which has no reward on top. As in SU1, but now E places one large cloth piece (15 cm × 20 cm) on one side and two small cloth pieces (15 cm × 12 cm and 15 cm × 8 cm) on the other side of the platform, so that the small piece is closer to S and the two pieces are visibly disconnected through a 1 cm gap. Rewards are placed at the same distance from S on each side, at the far ends of the 20 cm and 12 cm cloth pieces respectively. E scores whether S successfully retrieves the reward by pulling the largest cloth piece.

SU3: selecting between two cloth pieces under a bridge, pulling the cloth piece with a food reward on top of the cloth and under the bridge, instead of the cloth piece with a reward on top of the bridge. As in SU1, but now E places two identical cloth pieces (15 cm × 20 cm) on the two sides of the platform and two identical plastic bridges (21 cm × 3 cm × 6 cm) at the far ends of the two cloth pieces. One reward is placed on the top of one bridge, and another reward is placed on the cloth under the other bridge. E scores whether S successfully retrieves the reward by pulling the cloth with the reward on its top (if S chooses the cloth with the reward on the bridge, the cloth moves but the reward and the bridge remain stagnant).

SU4: selecting between two strings and pulling the long string with a food reward on top, instead of a combination of two short strings divided by a gap, the accessible of which has no reward on top. As in SU1, but now E places two ropes on the two sides of the platform, a longer rope (25 cm) already tied around a reward on the side further from S, and a shorter rope (15 cm) also tied to a reward on the side further from S. A small piece of rope (8 cm) is placed in front of the 15 cm rope, so that there is a 1 cm gap between the two. E scores whether S successfully retrieves the reward by pulling the longest rope.

SU5: selecting between two strings and pulling the long string with a food reward on top, instead of a combination of two short strings being slightly overlapping, the accessible of which has no reward on top. As in SU4, but now E places the shorter ropes so that the 8 cm rope is slightly but visibly overlapping the 15 cm rope (approximately 1 cm). E scores whether S successfully retrieves the reward by pulling the longest rope.

SU6: selecting between two strings and pulling the long string with a food reward on top, instead of a combination of two short adjacent strings, the accessible of which has no reward on top. As in SU4, but now E places the shorter ropes so that the 8 cm rope and the 15 cm rope are touching each other. E scores whether S successfully retrieves the reward by pulling the longest rope.

**MODELLING DETAILS**

Each task with a binomial response is parsed into a collection of binary values. The quantity y_ijt_ represents the result of the t^th^ trial for the i^th^ individual in the j^th^ task. If the j^th^ task has a rank response, the same notation may be used with the understanding that the trial number is constrained to equal 1. Bayesian models typically require specification of the likelihood of the observed responses (i.e., the y_ijt_’s) given the model parameters; classical statistical methods usually share this specification requirement in positing a model. Bayesian inference also requires an explicit mathematical representation of the prior, which reflects the uncertainty in all unknown quantities. The Bayesian approach is preferred to classical methods for these data for several reasons but especially because of the complexities with modelling rank data with a large number of ties; a characteristic of two tasks in the analysis data set.

The likelihood we used combined the data augmentation strategies of Albert & Chib [3] and Johnson and colleagues [4] to jointly model both binomial and rank data in a coherent manner. These strategies augment the observed data by introducing latent variables, and the latent variables and additional parameters introduce interpretive and computational advantages. To avoid an even more complex notation, we present the likelihood as though all *I* animals had completed all tasks (this only affects the indices over which products are taken). For a task *j* with a binomial response, the likelihood is given by

$$\prod_{i} \prod_{t} {1(z_{ijt}> \tau_{j})}^{yijt}{1(z_{ijt} \leq\tau_{j})}^{1-yijt},$$

where $\tau_{j}$ is a task-specific threshold parameter and *y_ijt_* equals 1 (0) if the trial resulted in a success (failure), an event that happens if and only if the latent variable for the trial exceeds the threshold; refer to Albert & Chib [3] for further details. For a task *j* with a rank response, the likelihood is given by

$$\left[ \prod_{i=1}^{I-1} p_{\left( ij \right)}(\kappa_{j}) \right]\left[ \prod_{i=1}^{I} \prod_{i^{'}:zi^{'}jt < zijt} 1(y_{i^{'}jt}\leq y_{ijt}) \right]$$

[see 4, equation 3). The first component is to model the probabilities of ties, and the second component is to ensure that the observed ranks concord with the ordering of the latent variables. For consecutive ordered latent variables, the ranks may either be tied or not; the probability they are tied is assumed to be an exponential function of the negative scaled difference between the latent variables [see 4, equation 2 for the definition of *p*_(_*_ij_*_)_(*κ*)].

Taking the product of the likelihood for each task produces the overall likelihood. This likelihood is the same for all of our models, regardless of the number of random effects used to model the latent variables. However, the prior distribution that we assumed for all unknown quantities differed across models. The most complex model we considered had species effects (*θ_s(i)_*), individual effects (*γ_i_*), species*domain effects (*ω_s(i),g(j)_*), individual*domain effects (*η_i,g(j)_*), and task-specific error variances (*σ_j_*^2^). The species to which the *i*^th^ individual belongs is represented by *s(i)* and the domain to which the *j*^th^ task belongs is represented by *g(j)*. For the prior specification of the most complex model, we assumed that:

$z_{ijt}| \left( \theta_{s\left( i \right)}, \gamma_{i}, \omega_{s\left( i \right),g\left( j \right)}, \eta_{i,g\left( j \right)}, \sigma_{j}^{2} \right) \sim N(\theta_{s\left( i \right)}+ \gamma_{i}+ \omega_{s\left( i \right),g\left( j \right)}+ \eta_{i,g\left( j \right)}, \sigma_{j}^{2}$)

$\theta_{s\left( i \right)}| \sigma_{\theta}^{2} \sim N(0, \sigma_{\theta}^{2}$)

$\gamma_{i}| \sigma_{\gamma}^{2} \sim N(0, \sigma_{\gamma}^{2}$)

$\omega_{s\left( i \right),g\left( j \right)}| \sigma_{\omega}^{2} \sim N(0, \sigma_{\omega}^{2}$)

$\eta_{i,g\left( j \right)}| \sigma_{\eta}^{2} \sim N(0, \sigma_{\eta}^{2}$)

$$\sigma_{j}^{2}|\sigma_{\varepsilon}^{2} \sim Inverse Gamma(10.782, 9.782\sigma_{\varepsilon}^{2})$$

(*σ_ε_*^2^, *σ_θ_*^2^, *σ_γ_*^2^, *σ_ω_*^2^, *σ_η_*^2^) *~ Dirichlet (1.425, 1.425, 1.425, 1.425, 1.425)*

*τ_j_ ~ Cauchy(0, 0.5^2^)*

*κ_j_ ~ Exponential(1)*

The prior distribution for each *σ_j_*^2^| *σ_ε_*^2^ was chosen to have the *a priori* average value of each task’s error variance be the same across tasks and to have a prior probability of 0.8 that any given *σ_j_*^2^| *σ_ε_*^2^ was in (2*σ_ε_*^2^/3, 3*σ_ε_*^2^/2). This permits the error variances to differ across tasks but assumes that most of them were likely to be similar. The prior distribution of the five variance components (*σ_ε_*^2^, *σ_θ_*^2^, *σ_γ_*^2^, *σ_ω_*^2^, *σ_η_*^2^) was chosen based on three criteria: the overall variance of the latent variables was constrained to equal 1 as an identifying restriction; each included component had the same prior marginal distribution; and the prior probability that any given component was less than 1/25^th^ of its expected value (less than 1/125 because there are five components) was assumed to equal 0.01. The second and third criteria were included so that the relative importance of all effects in the model was considered to be the same, avoiding a preference for one effect above another. The distributions of the *τ_j_*’s and the *κ_j_*’s were chosen based somewhat on characteristics of the data, but they were nonetheless fairly vague, were common to all models considered, and were not of primary interest. Therefore, the dependence of the latter distributions on the data was not worrisome.

In the other models considered, the prior specification followed the same guidelines. For every random effect that was excluded, there was one less variance component. Consequently, the values in the Dirichlet distribution were adjusted to again satisfy the third criterion mentioned in the previous paragraph. For those models that restricted the error variance to be identical for all tasks, the inverse gamma prior was replaced with the requirement that *σ_j_*^2^| *σ_ε_*^2^ must exactly equal *σ_ε_*^2^.

The prior distributions were not particularly informative and there was a large quantity of data. Therefore, the effect of the prior on the final conclusions for our primary questions was minor. We emphasize that the Bayesian analysis was merited because of its ability to both seamlessly handle rank data with many ties and to incorporate hierarchical effects. We did not include strong prior beliefs, which are often a motivating factor for conducting Bayesian analyses. Nonetheless, to assess the sensitivity of our results to our prior specification, we removed the third criterion mentioned in the penultimate paragraph and substituted the value 0.25 for each parameter of the Dirichlet distribution. That is, we modified the prior distribution to reflect *very* little prior knowledge about the values of the variance parameters, but still assumed *a priori* that they each were equally important. The proportion of variance due to each effect in the best model (with only species and species*domain effects) changed only slightly compared to the initial prior specification: error was assumed to account for 70% of the variability in the latent performance variables (up from 66%); species effects were assumed to account for 13% of the variability (down from 17%); and species*domain effects were assumed to account for 17% of the variability (stable at 17%). Our results are seen to be very similar under each of these alternatives, and we present detailed results only for the original prior specification.

**REFERENCES**

1. Kappeler PM, Pereira ME (2003) Primate life histories and socioecology. Chicago: Chicago University Press. 395 p.
2. Smuts BB, Cheney DL, Seyfarth RM, Wrangham RW, Struhsaker TT (1987) Primate societies. Chicago: Chicago University Press. 578 p.
3. Albert JH, Chib S (1993) Bayesian analysis of binary and polychotomous response data. J Amer Statist Assoc 88:669-679.
4. Johnson VE, Deaner RO, van Schaik CP (2002) Bayesian analysis of rank data with application to primate intelligence experiments. J Amer Statist Assoc 97:8-17.
